# Looking for “fNIRS Signature” in Autism Spectrum: A Systematic Review Starting From Preschoolers

**DOI:** 10.3389/fnins.2022.785993

**Published:** 2022-03-02

**Authors:** Eugenia Conti, Elena Scaffei, Chiara Bosetti, Viviana Marchi, Valeria Costanzo, Valerio Dell’Oste, Raffaele Mazziotti, Liliana Dell’Osso, Claudia Carmassi, Filippo Muratori, Laura Baroncelli, Sara Calderoni, Roberta Battini

**Affiliations:** ^1^Department of Developmental Neuroscience, IRCCS Stella Maris Foundation, Pisa, Italy; ^2^Department of Neuroscience, Psychology, Drug Research and Child Health NEUROFARBA, University of Florence, Florence, Italy; ^3^Department of Clinical and Experimental Medicine, University of Pisa, Pisa, Italy; ^4^Institute of Neuroscience, National Research Council, Pisa, Italy

**Keywords:** fNIRS, near-infrared spectroscopy, functional neuroimaging, preschooler, neurodevelopmental disorders, autism spectrum disorder, high-risk infant

## Abstract

Accumulating evidence suggests that functional Near-Infrared Spectroscopy (fNIRS) can provide an essential bridge between our current understanding of neural circuit organization and cortical activity in the developing brain. Indeed, fNIRS allows studying brain functions through the measurement of neurovascular coupling that links neural activity to subsequent changes in cerebral blood flow and hemoglobin oxygenation levels. While the literature offers a multitude of fNIRS applications to typical development, only recently this tool has been extended to the study of neurodevelopmental disorders (NDDs). The exponential rise of scientific publications on this topic during the last years reflects the interest to identify a “fNIRS signature” as a biomarker of high translational value to support both early clinical diagnosis and treatment outcome. The purpose of this systematic review is to describe the updating clinical applications of fNIRS in NDDs, with a specific focus on preschool population. Starting from this rationale, a systematic search was conducted for relevant studies in different scientific databases (Pubmed, Scopus, and Web of Science) resulting in 13 published articles. In these studies, fNIRS was applied in individuals with Autism Spectrum Disorder (ASD) or infants at high risk of developing ASD. Both functional connectivity in resting-state conditions and task-evoked brain activation using multiple experimental paradigms were used in the selected investigations, suggesting that fNIRS might be considered a promising method for identifying early quantitative biomarkers in the autism field.

## Introduction

Functional Near-Infrared Spectroscopy (fNIRS) allows performing *in vivo* a continuous and non-invasive monitoring of oxygenation levels of chromophores like hemoglobin (Van de Rijt et al., 2018), thanks to the transparency of biological tissues -including the human brain- to the light in the near-infrared spectrum. Indeed, fNIRS quantifies regional changes in the concentration of oxygenated, deoxygenated, and total hemoglobin (OxyHb, DeoxyHb, and TotHb). The blood-oxygen-level-dependent (BOLD) signal detected by fNIRS provides a direct measure of modifications in the brain blood flow, which are coupled to neuronal activity by the complex process known as “neurovascular coupling” (Villringer and Chance, 1997; Logothetis and Wandell, 2004). Typically, an increase of neural activity is paralleled by a peak of OxyHb and TotHb, with a drop of DeoxyHb. fNIRS devices consist of red light-sources paired to specific detectors, which can be placed into a textile EEG-like cap to form an array of multi-distant channels. For data acquisition, the system executes the parallel reading of optical sensors. fNIRS systems give the opportunity to perform recording with different wavelengths of the near-infrared spectrum (700–1000 nm). However, for functional activation studies in humans the devices typically focus on paired wavelengths of approximately 690 and 830 nm to optimize the accuracy of OxyHb and DeoxyHb measurements (Lloyd-Fox et al., 2010). Indeed, separability between the chromophore signals seems to be optimal if one wavelength is below 720 nm and the other is above 730 nm (Uludag et al., 2004).

Although several neuroimaging methods can directly detect the electrical activity of brain circuits (EEG and MEG) or record the related hemodynamic response (fMRI), most of them have limiting factors restricting their use in the developmental time window (for instance, sensitivity to motion artifacts, requiring high grade of subject compliance). Indeed, fMRI is the gold standard for *in vivo* imaging of the human brain, but fNIRS stands out for its high portability, robustness to noise, relatively low costs and small size, bringing functional imaging into much more realistic environments (Cui et al., 2011; Duan et al., 2012; Scarapicchia et al., 2017). In particular, strength to motion artifacts make this technique an ideal candidate for research in preschool children (until the age of 6), even in infancy and toddlerhood (Lloyd-Fox et al., 2010). Despite lower spatial resolution and imaging depth, fNIRS offers a higher temporal resolution than fMRI. On the other hand, EEG and fNIRS share high portability, low-cost features and recording depth, but they measure different perspectives of brain activity: indeed, EEG signal reflects the bioelectrical activation of cortical neurons, while fNIRS assesses hemodynamic responses (Chiarelli et al., 2017; Berger et al., 2019). In order to obtain a more detailed picture of brain activity, fNIRS can be combined with other neuroimaging and electrophysiological methods (e.g., fMRI and EEG), without causing any measurement interference (Chen et al., 2015) and potentially gaining a multimodal data set (Yuan et al., 2010; Chiarelli et al., 2017). Notably, EEG and fNIRS use similar experimental settings (they are both scalp-located procedures), but show different temporal resolutions that might allow dissecting the electrical from the hemodynamic contribution to the recorded signal.

Although introduced into clinical care almost 40 years ago, only recently, fNIRS gained much popularity in the study of brain development and neurodevelopmental disorders (NDDs) (Greisen, 2006; Vanderwert and Nelson, 2014). FNIRS has been widely used to investigate the typical maturation of speech perception and language development, sensory and motor functions, social communication and interaction, object processing, human action processing in both toddlers and children (Lloyd-Fox et al., 2010). In contrast, the application of fNIRS in the field of NDDs is a growing research area and studies looking for a “fNIRS signature” as a brain biomarker useful for the diagnosis and the treatment outcome are still underrepresented (Gervain et al., 2011). NDDs are a heterogeneous group of complex disorders resulting from the interaction of genetic, epigenetic, neurobiological and environmental factors (from alterations *in utero* to postnatal environment), and characterized by early disruption in cognition, emotion, and behavior. NDDs include attention-deficit/hyperactivity disorder (ADHD), autism spectrum disorder (ASD), communication and specific learning disorders, intellectual disability (ID) and motor disorders (American Psychiatric Association, 2013). NDDs often present overlapping clinical features or may occur simultaneously in the same patient, making the differential diagnosis potentially challenging (Hansen et al., 2018). Moreover, NDDs show fairly broad behavioral phenotypes, reflecting also etiological heterogeneity of these disorders that can occur as “idiopathic” or “secondary” conditions (e.g., well documented causes of secondary ASD include monogenic disorders such as tuberous sclerosis and Fragile-X syndrome) (Muhle et al., 2004). Thus, reliable functional biomarkers would be very helpful to support early clinical diagnosis and to monitor developmental trajectories through *in vivo* longitudinal studies, also focused on the potential outcome of tailored intervention. Moreover functional biomarkers might help clinicians to separate behavioral symptoms of NDDs into more stable and objective phenotypes, including gender-related endophenotypes.

Clinically useful neuroimaging markers for NDDs are sorely missing yet (Vasung et al., 2019; Azhari et al., 2020), but emerging evidence suggests that fNIRS might be exploited to generate unbiased and reliable measures to explore brain functions, thus possibly driving clinicians toward a faster and more precise diagnosis (Zhang and Roeyers, 2019).

Moreover, fNIRS offers the opportunity to study cortical networks focusing not only on neuronal activation, but also on the neuromodulatory action of non-neuronal cells (such as glial cells or astrocytes that contribute to neurovascular and metabolic homeostasis of the brain). Thus, fNIRS is emerging as a fundamental tool, alone or in combination with other techniques, to dissect the key players in brain physiology and pathophysiology (Lines et al., 2020).

The main purpose of this systematic review has been to investigate the recent literature covering the use of fNIRS in the field of NDDs. We decided to focus on the preschool population in order to highlight the promising value of fNIRS as a reliable and non-invasive tool to detect brain biomarkers in clinical settings, even in populations commonly showing reduced compliance to experimental environments. This specific inclusion criterium led to narrow the focus of this review to children with idiopathic ASD or high-risk (HR) infants.

## Materials and Methods

A systematic search was conducted for relevant studies in three databases (PubMed, Scopus, and Web of Science), using the following search terms that were refined from previous reviews (Liu et al., 2019; Zhang and Roeyers, 2019) and according to PICOS framework: (“fNIRS” OR “functional nirs” OR “near-infrared spectroscopy”) AND (“neurodevelopmental disorder” OR “ASD” OR “ADHD”) AND (“children” OR “toddler” OR “preschooler”).

Additionally, manual searches were conducted among the reference sections of the retrieved studies and reviews. Publication year was not restricted, and the latest database search was performed in March 2021. Reviews were not included in the selection, but were only used to collect original studies. After removing duplicate records, articles emerged from the search were required to meet pre-established criteria. Inclusion and exclusion criteria, established prior to the literature search, are outlined below.

### Inclusion Criteria

(i) Studies applying fNIRS;

(ii) Studies including a clinical population with a confirmed diagnosis (according to DSM-5) or a well-established condition of risk for NDDs;

(iii) Studies including preschool children (sample mean age < 7 years).

### Exclusion Criteria

(i) Articles published in languages other than English;

(ii) Meta-analyses or literature reviews;

(iii) Other reasons referring to report characteristics, as not published clinical studies (e.g., conference paper, thesis, book chapter) or studies that do not involve human participants (e.g., focused on methodological issues).

## Results

### Study Selection

**FIGURE 1:**
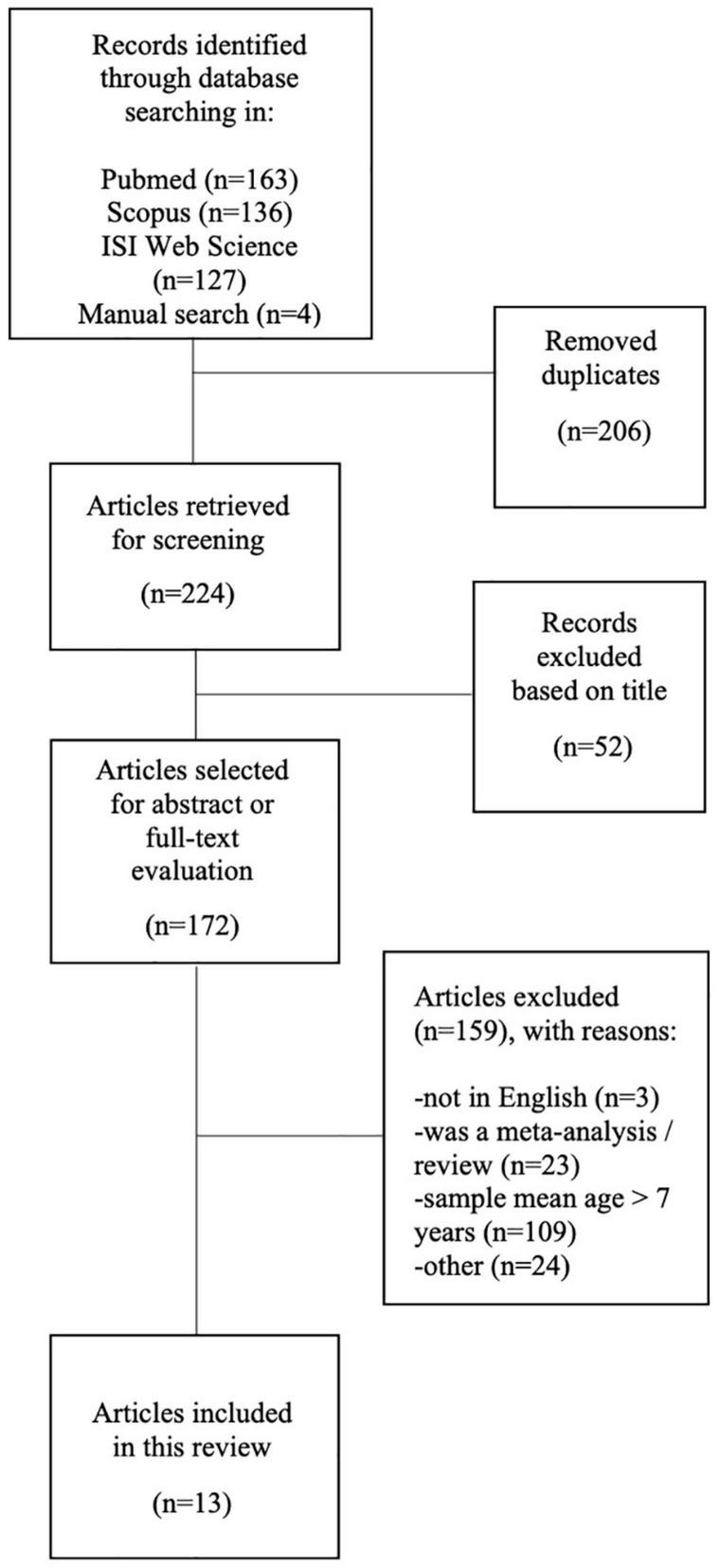
Flow diagram of the literature search and subsequent screening performed in this review.

One hundred and sixty-three potentially relevant studies were identified from PubMed, 136 from Scopus, 127 from Web of Science; in addition, four articles that met the selection criteria were retrieved from screened review articles. After omitting duplicates, 224 articles were examined. On the basis of the title, a total of 52 studies that did not meet the inclusion criteria were disregarded. Finally, within the172 articles screened for abstract or full-text evaluation, a total of 13 studies were selected for this review. An in-depth detail of paper selection is shown in the PRISMA diagram (Figure 1).

Crucial information was extrapolated from included papers. Tables 1, 2 summarize the mean age of participants, the sample size and gender ratio, the experimental procedures (technical aspects of recording sessions and experimental design) used during fNIRS measurement.

**TABLE 1:** Overview of the reviewed studies using Resting State (R-S) fNIRS recordings: references, technical issues, characteristics of participants as well as main findings are shown.

References	NIRS technique			Experimental paradigm	Characteristics of participants		Main findings
First Author, year	System	Channels (N)	Probe Geometry Source: Detector (cortical areas covered)	Experimental setting “trick” for Resting-State recording	Clinical population N (M:F) Mean age	Comparison group N (M:F) Mean age	
Kikuchi et al., 2013	Foire 3000, (Shimadzu Kyoto, Japan)	2	2:1 optodes probe (covering anterior prefrontal cortex, bilaterally)	R-S fNIRS measurement while watching picture-card show	ASD 15 (13:2) 45–82 months^	TD 15 (13:2) 47–86 months^	Higher anterior prefrontal cortex (PFC) connectivity for 0.02-Hz fluctuations in children with ASD compared to TD group. Inter-hemispheric connectivity in ASD group positively correlated with the severity of social deficit.
Li and Yu, 2016	LABNIRS (Shimadzu Corporation, Kyoto, Japan)	44	16:16 optodes probe (covering prefrontal, temporal and occipital areas, bilaterally)	R-S fNIRS measurement while watching cartoon	ASD 12 (9:3) 6.1 years	TD 12 (9:3) 6.1 years	Weak functional network efficiency in young children with ASD compared to TD group. In particular, weak lobe-level inter – region connectivity in right prefrontal cortex (including its linkages with left prefrontal cortex and bilateral temporal cortex) was found.
Li and Yu, 2018	LABNIRS (Shimadzu Corporation, Kyoto, Japan)	44	16:16 optodes probe (covering prefrontal, temporal and occipital areas, bilaterally)	R-S fNIRS measurement while watching cartoon	ASD 46 (36:10) 5 years	n.a.	Significant associations between age and network efficiency, especially evident in the deoxy- and total-Hb-based-networks, indicating the network efficiency decreases with age in young ASD children. Significant associations between levels of autistic behaviors and network efficiency in the oxy-Hb-based-network, indicating decreased functional global and local network efficiency in ASDs with a relatively higher level of autistic behaviors.
Li et al., 2018	LABNIRS (Shimadzu Corporation, Kyoto, Japan)	44	16:16 optodes probe (covering prefrontal, temporal and occipital areas, bilaterally)	R-S fNIRS measurement while watching cartoon	ASD 29 (23:9) 6 years	TD 29 (20:9) 6.5 years	Spatial complexity of functional connectivity (SCFC) differs between ASD and control group. Global SCFC was significantly higher in ASDs, along with considerably higher intraregion SCFCs in the prefrontal and temporal lobes. Moreover, higher interregion SCFCs between right PFC and the other brain regions were found in young children with ASD compared to TD children.
Jia et al., 2018	LABNIRS (Shimadzu Corporation, Kyoto, Japan)	44	16:16 optodes probe (covering prefrontal, temporal and occipital areas, bilaterally)	R-S fNIRS measurement while watching cartoon	ASD 35 (23:12) 5.9 years	TD 31 (20:11) 6.6 years	The long-range temporal correlations (LRTCs) of hemoglobin concentration signals were attenuated in young children with ASD over left temporal region (for oxy-Hb signal) and bilateral temporo-occipital regions (for deoxy-Hb signal). Detrended fluctuation analysis (DFA), used to quantify LRTCs, of oxy-Hb in left temporal region were negatively correlated with autistic symptom severity. Relationship between age and LRTCs of Hb concentration signals differs between two groups, correlating with autistic symptom severity.

*^ Sample mean age not available: age range of participants is shown.*

*N.A. means not applicable: no comparison group is included in this study.*

**TABLE 2:** Overview of the reviewed studies using Task-Related (T-R) fNIRS recordings: references, technical issues, characteristics of participants as well as main findings are shown.

References	NIRS technique			fNIRS paradigm	Demographics		Main findings
First Author, year	System	Channels (N)	Probe Geometry Source: Detector (cortical areas covered)	Type of stimuli proposed in Task-Related (T-R) protocol	Clinical population N (M:F) Mean age	Comparison group N (M:F) Mean age	
Fox et al., 2013	Hitachi ETG-4000 system	24	Two arrays of optodes probe: -Frontal areas (OFC), bilaterally (Ch 1-12) -Temporal lateral areas, only in right hemisphere (Ch 13-24)	Face processing task	HR 10 (4:6) 7 months	LR 10 (4:6) 6.9 months	Greater OxyHb responses in lateral regions for LR as compared to HR, and significantly greater DeoxyHb responses in frontal channels for HR as compared to LR during face processing task. Greater DeoxyHb response to mother’s face as opposed to stranger face within the HR group across both frontal and lateral regions. Greater OxyHb response to smiling versus neutral conditions only for the LR.
Lloyd-Fox et al., 2013	UCL-NIRS topography system	26	10:10 optodes probe (covering temporal areas, as IFG, pSTS-TPJ and aMTG-STG, bilaterally)	Social perception task	HR 18 (8:10) * 5 months	LR 16 (10:6) * 5 months	Visual social stimuli produced a diminished response in HR infants relative to LR infants, with difference mostly evident in the left STS region of the cortex. Regarding auditory social stimuli, no vocal-specialized areas in HR group was found, compared to greater vocal selectivity in the right mid-posterior STS region in LR group.
Lloyd-Fox et al., 2018	UCL-NIRS topography system	26	10:10 optodes probe (covering temporal areas, as IFG, pSTS-TPJ and aMTG-STG, bilaterally)	Social perception task	HR^ 20 (10:10) * 5 months	LR 16 (10:6) * 5 months	Early fNIRS measurements (4–6 months) in response to social perception task correlate with later clinical outcome and symptoms level at 36 months. Reduced activation to visual social stimuli in those infants with later diagnosis of ASD. Reduced activation to vocal relative to non-vocal sounds in those infants with later diagnosis of ASD compared to LR and HR-without later diagnosis confirmation.
Braukmann et al., 2018	UCL-NIRS topography system	26	10:10 optodes probe (covering temporal areas, as IFG, pSTS-TPJ and aMTG-STG, bilaterally)	Social perception task	HR 16 (7:9) 5 months	LR 13 (9:4) 5 months	LR showed a socially selective cortical response in the right posterior temporal cortex in response to visual social stimuli, whereas this response was not significant in the HR.
Bhat et al., 2019	Hitachi ETG-4000 system	24	10:8 optodes probe (covering temporal areas, bilaterally)	Naturalistic social interactions	HR 9 (7:2) 7 months	LR 6 (1:5) 7.5 months	HR infants showed reduced right and left-hemispheric activation compared to LR infants based on OxyHb and DeoxyHb signal trends. Indeed, HR infants had greater functional connectivity than LR infants during the pre- and post-social periods and showed a drop in connectivity during the social period.
Keehn et al., 2013	Hitachi ETG-4000 system	24	10:8 optode probe (covering anterior and posterior temporal areas, bilaterally)	Language-related task	HR 27°	LR 37°	In a prospective longitudinal view, LR infants showed a pattern of increasing functional connectivity from 3- to 12-months in selected ROIs, while HR exhibited a pattern of decreasing connectivity with age. At 12-months, HR have reduced intra-hemispheric connectivity for the left hemisphere compared to LR infants.
Edwards et al., 2017	Hitachi ETG-4000 system	24	10:8 optode probe (covering anterior and posterior temporal areas, bilaterally)	Language-related task	HR 21 (13:8) 3.6 months	LR 17 (10:7) 3.6 months	During an auditory stimuli containing syllable repetitions (ABB vs. ABC syllables pattern), HR females exhibited significantly higher OxyHb responses than HR males, LR females or LR males in both left and right anterior regions. HR females exhibited different temporal neural response patterns than LR females across both ABB and ABC stimuli pattern (no habituation response to repetition in speech in HR females group).
Pecukonis et al., 2021	Hitachi ETG-4000 system	24	10:8 optode probe (covering anterior and posterior temporal areas, bilaterally)	Language-related task	HR^ 14 (7:7) 7 months	LR 18 (9:9) 7 months	LR infants exhibited strongest activation in bilateral anterior ROIs, while HR exhibited similar activation across all brain regions in study. Compared to LRs, HR-ASD infants had reduced brain response in the bilateral anterior ROIs, while HR-noASD had increased brain response in the right posterior ROI. This atypical brain response was not predictive of 24-month language abilities in HR infants.

*Note that 32 of the 36 infants (16 low risk and 16 high risk) contributed data to a previous publication (Lloyd-Fox et al., 2013).

*°Longitudinal Study: 3-, 6-, 9-, and 12- months visit; variable gender ratio according to timepoint.*

*^Longitudinal data outcome available: within HR cohort of Lloyd-Fox et al. (2018) at 36-months visit 15 HR-noASD and 5 HR-ASD; within HR cohort of Pecukonis et al. (2021) at 36-months visit 9 HR-noASD; 5 HR-ASD.*

Although the initial aim was to include different type of NDDs (see keywords used for the search), only studies focused on children with idiopathic ASD or high-risk (HR) infants (infants at high familial risk of autism) satisfied the inclusion and exclusion criteria.

Regarding other NDDs, studies on ADHD included children older than 7 years, due to the pharmacological aim of their experimental design: at the time of our last search, indeed, 55 studies focused on fNIRS and ADHD, but none of them was performed on the preschool population. Moreover, within the growing bulk of literature that uses fNIRS for evaluating the emerging language ability in infants, only two studies (Chang, 2014; Hosseini et al., 2018) focused on children with Language Disorders; however, both of them, did not meet the inclusion criteria (mean age of participants older than 7 years) and were therefore excluded from this review. fNIRS was rarely used to explore neuropsychological features in learning disorders such as Dyslexia (Zhu et al., 2012; Pecyna and Pokorski, 2013; Song et al., 2013; Sela et al., 2014; Cutini et al., 2016), but this field obviously includes only school-aged children. Application of fNIRS to Intellectual Disability (ID) is only anecdotal yet (only one scientific report found in literature database consulted) (Bembich et al., 2021).

### Characteristics of Participants

As mentioned above, all studies included in this review applied fNIRS in preschool children with ASD, a clinical condition characterized by socio-communicative impairment associated with restricted interests and repetitive behaviors, or HR infants (younger siblings of children with ASD). Only four studies included more than 40 participants (Keehn et al., 2013; Jia et al., 2018; Li and Yu, 2018; Li et al., 2018); in most cases subjects were equally distributed between the clinical population and the control group. Interestingly, the cohorts included both males and females in all studies, but the two populations were not homogeneously distributed in terms of sample size, except for two investigations (Lloyd-Fox et al., 2018; Pecukonis et al., 2021).

Five studies included in this review (Kikuchi et al., 2013; Li and Yu, 2016, 2018; Jia et al., 2018; Li et al., 2018) comprised ASD children with a mean age was between 3 and 7 years and compared the ASD group with age- and gender-matched typically developing (TD) peers in resting state condition (see Table 1). It is to note that all selected studies included children with an average IQ score and no comorbidity with ADHD.

The remaining eight studies (Fox et al., 2013; Keehn et al., 2013; Lloyd-Fox et al., 2013, 2018; Edwards et al., 2017; Braukmann et al., 2018; Bhat et al., 2019; Pecukonis et al., 2021) focused on infants/toddlers (children younger than 3 years old), and compared fNIRS data between HR group and in Low-Risk groups (LR; condition defined as subjects with no family history of ASD) in task-related study designs (see Table 2). Of these studies, only two (Lloyd-Fox et al., 2018; Pecukonis et al., 2021) analyzed the correlation between the fNIRS brain responses of infants during the first year of life and their diagnostic outcome at 24 or 36 months.

### fNIRS Measurement: Technical Issues and Experimental Design

A total of four different fNIRS recording systems were employed in the reviewed studies (Foire 3000, LABNIRS, Hitachi ETG-4000 system, UCL-NIRS topography system). The number of channels used ranged from a minimum of 2 (Kikuchi et al., 2013) to a maximum of 44 (Li and Yu, 2016, 2018; Jia et al., 2018; Li et al., 2018). The remaining investigations used an intermediate number of channels (24–26 pairs of optodes), but the density of montages may be considered comparable among almost all studies, due to the younger age of the cohort investigated and the relatively smaller dimension of the probe. Despite the variability of probe geometry, in all cases optodes placement followed the international 10–20 EEG system.

Within the 13 articles included, eight studies explored the brain activation evoked by specific tasks (task-related paradigm or T-R; an overview of experimental procedures is reported in Table 2) and the remaining five investigated functional brain organization in resting-state (R-S) condition (an overview of experimental procedures is reported in Table 1). Considering the significant difficulties to remain still under stimulation-free conditions for young children, all resting-state data were recorded while participants watched a cartoon or, only in one case, a picture-card show (Kikuchi et al., 2013) in order to optimize subject compliance. Papers exploring functional cortical activation in task-related condition can be sub-grouped into two categories that reflect the nature of the task proposed: (i) social stimuli, including social perception (Lloyd-Fox et al., 2013, 2018; Braukmann et al., 2018), face processing (Fox et al., 2013) and naturalistic social interactions (Bhat et al., 2019); (ii) language-related stimuli (Keehn et al., 2013; Edwards et al., 2017; Pecukonis et al., 2021).

## Discussion

Papers systematically selected for this review allow discussing some research questions that might pave the way toward the fNIRS application in the field of NDDs. The final selection, mainly based on age criteria, focused our attention on ASD and HR populations. This field is characterized by huge heterogeneity both regarding the phenotypic and the neurobiological level, and a great amount of literature is devoted to uncover biomarkers dissecting sub-phenotypes and supporting the clinical assessment (Loth et al., 2016; Lombardo et al., 2019). Indeed, while an early detection of ASD is highly recommended to receive the services and supports the children need to reach their full potential (Dawson et al., 2010; Hyman et al., 2020), a final diagnosis is still obtained approximately during the third year of life (Salomone et al., 2016). However, neuroimaging and electrophysiological evidence consistently suggested that ASD-related atypical brain pattern in cortical regions crucial for socio-communicative skills (such as fronto-temporal areas) could be detected before the full-blown expression of symptoms (Conti et al., 2015; Ha et al., 2015; O’Reilly et al., 2017; Pagnozzi et al., 2018).

### Is “fNIRS Signature” Useful as a Brain Biomarker for Autism Spectrum Disorder?

Resting-state fNIRS measurements have been proposed as auxiliary indexes for the objective assessment of ASD in both young (Xu et al., 2021) and adult population (Yanagisawa et al., 2016).

Regarding preschoolers, Kikuchi et al. (2013) first demonstrated an aberrant functional connectivity between the right and the left anterior prefrontal cortex (aPFC) in young children with ASD under resting state conditions. Notably, they found a significantly higher inter-hemispheric connectivity in the ASD compared to the TD group, reinforcing previous data about the aberrant cortical organization at structural level in ASD children (McCaffery and Deutsch, 2005; Courchesne et al., 2011). The higher inter-hemispheric connectivity in ASD reported by Kikuchi et al. (2013) was positively correlated with the severity of social deficit, as scored with the Autism Diagnostic Observation Schedule (Lord et al., 2012). It is to note that the anterior prefrontal cortex has already been implicated in ASD pathophysiology in previous structural and functional studies, as being involved in social perception and processing abilities that are altered in the ASD condition (Uddin et al., 2013; Pagnozzi et al., 2018).

In contrast, studies from Li and Yu (2016, 2018) and Li et al. (2018) showed weaker functional network efficiency in young children with ASD compared to TD peers, mainly in short- and long-range connectivity of the right prefrontal cortex (Li and Yu, 2016). Moreover, a subsequent study of the same research group (Li et al., 2018) showed an inverse association between network efficiency, age (especially evident in the Deoxy- and TotHb-based networks) and severity of autistic behaviors (especially evident in the OxyHb-based-network) in high-functioning ASD subjects. Statistically significant findings were also obtained when comparing ASD and TD groups through spatial complexity of functional connectivity (SCFC) analysis (Li et al., 2018).

Other approaches to analyze resting state recordings also confirmed that fNIRS might be a candidate tool to investigate the pathophysiological mechanism of ASD, looking at the temporal dynamics of neuronal oscillations (Jia et al., 2018). Specifically, the long-range temporal correlations (LRTCs) of hemoglobin concentration signals were studied by quantifying a detrended fluctuation analysis (DFA) exponent. Comparing data between ASD and TD group, significant differences were found over the left temporal region for OxyHb signal, and over bilateral temporo-occipital regions for DeoxyHb signals, suggesting a shift-to-randomness of brain oscillations in children with ASD. Moreover, the relationship between age and DFA exponents revealed that this association could be modulated by autism. Furthermore, the DFA exponents of OxyHb in the left temporal region were negatively correlated with autistic symptom severity. Between-group differences remained significant also looking at correlation coefficients between age and DFA.

Altogether, these results confirm that fNIRS can detect a different pattern of functional connectivity in the brain of ASD children. Accordingly, the considerable bulk of prospective studies focused on neurostructural and neurofunctional measures of brain development validated the concept of ASD as characterized by early altered connectivity patterns (Bosl et al., 2011; Wolff et al., 2012; Solso et al., 2016). In particular, early social brain network alterations were previously reported in structural (Shen et al., 2018) and functional MRI (Emerson et al., 2017) as well in observational studies (Elsabbagh and Johnson, 2016). However, no resting-state fNIRS data are yet available in cohorts younger than 3 years, thus restricting the predictive value of resting state analyses so far. Moreover, the mathematical algorithms used for the interpretation of resting-state data are very complex to be applied in the clinical setting and the limited coherence among models still prevents from drawing conclusive interpretations.

### Is “fNIRS Signature” Useful as an Early Predictor in “At Risk” Population?

Younger siblings of children with ASD are considered as HR infants because around 20% of them receive a diagnosis of ASD at the age of 3 years (Ozonoff et al., 2011) and a further 20–30% exhibit other neurodevelopmental problems (Messinger et al., 2013). In addition, a high rate of behavioral traits overlapping with those observed in children with an ASD diagnosis (referred as the Broader Autism Phenotype) has been documented in the sibling population (Charman et al., 2017). Thus, the prospective study of the HR population allows detecting behavioral risk signs or biomarkers of NDDs at a very early age, prior to the full clinical manifestation (Zwaigenbaum et al., 2015).

Studies on younger siblings of children with ASD compared HR infants with infants without familial history of ASD or NDDs (LR infants), regardless their developmental outcome or differentiating HR infants with or without a later diagnosis of ASD. In this review, we included both types of studies as we are interested in understanding how fNIRS signal can be used as a potential biomarker of atypical development, and not only as an ASD related outcome.

In the past decade, the number of studies exploring the applicability of fNIRS technique in HR infants has rapidly grown, focusing especially on core ASD neurocognitive domains. Accordingly, our study selection includes five papers testing social perception and three others assessing possible alterations of language development in the HR group.

With regard to social processing, starting from previous data in typically developing infants (Lloyd-Fox et al., 2012), Lloyd-Fox et al. (2013) examined whether the temporal lobe specialization for processing visual and auditory social stimuli during the first months of life differed between HR and LR infants. They found significantly diminished evoked hemodynamic responses in HR compared to LR infants in the superior temporal sulcus (STS), both in visual and auditory trials, suggesting a lack of cortical specialization to social stimuli, already within the first 6 months of life. Notably, a more recent prospective study in the same cohort of HR infants (increased by only 2 more subjects) demonstrated that the reduced activation to visual social and vocal stimuli across cortical regions of interest was specific for infants with a later diagnosis of ASD (Lloyd-Fox et al., 2018).

Moreover, Braukmann et al. (2018) provided further evidence for a social processing difference in infants at risk of autism, highlighting a reduced hemodynamic response evoked by social visual stimuli in the right posterior temporal cortex. Similarly, a reduced bilateral cortical activation in HR children compared to LR infants was reported in the social perception within the infant-mother dyad (Bhat et al., 2019). Of note, this is the only study using a fNIRS experimental protocol with live stimuli (face-to-face naturalistic social interaction between infant and mother).

Social behavior was also explored by Fox et al. (2013), recording fNIRS hemodynamic responses to a face perception task (facial identity and emotion perception on video clips performed by infant’s mothers) during the first year of life. This study suggested differences in patterns of functional connectivity in the frontal lobe (short- and long-range connections) between the HR and LR groups. HR infants showed significantly greater DeoxyHb responses in frontal channels and lower OxyHb responses in right temporal regions during face processing task; a significant difference between groups was detectable in response to mother’s face as to a stranger face (greater DeoxyHb response within the HR group across both frontal and lateral regions). For this cohort, no longitudinal data were available for discriminating infants with or without later ASD diagnosis and establishing the predictive value of early fNIRS recordings.

To summarize, these results suggest that socially evoked fNIRS signals might be a suitable and predictive biomarker in early assessment of HR infants; however, further studies are needed to confirm these promising findings.

Growing evidence on the validity of fNIRS for studying early aberrant functional brain networks in HR infants emerged from the language domain as well.

Keehn et al. (2013) explored functional connectivity during the first year of life in HR infants using a task-related experimental paradigm (language processing task). They found that from 3 to 12 months HR children exhibited a pattern of decreasing connectivity with reduced intra-hemispheric connectivity for the left hemisphere compared to LR infants at 12 months.

Moreover, an alteration of language processing can be detected very precociously with fNIRS: Edwards et al. (2017) analyzed precursors of language development in HR and LR 3-month-old infants: they found that HR females exhibited significantly higher OxyHb responses to auditory stimuli containing syllable repetitions than HR males, LR females or LR males in both left and right anterior brain regions. It is worth noting that HR females showed different temporal response patterns with respect to LR females across different type of auditory pattern (repeating vs. non-repeating syllabic sequences) presented randomly (speech-like stimuli), indicating a potential gender endophenotype in ASD. Using the same experimental protocol, Pecukonis et al. (2021) performed a longitudinal study on functional specialization of language-related brain regions during the first year of life in HR and LR group, suggesting a possible role of language-evoked fNIRS measurements to predict the diagnostic outcome of infants at 24 months.

Altogether, these data demonstrate that fNIRS studies on siblings can provide a unique window into the earliest neurobiological atypical trajectories and have the potential to shed light on possible ASD-related endophenotypes and biomarkers of brain function. In particular, studies correlating fNIRS brain responses in the first year of life to the diagnostic outcome at 24–36 months (Lloyd-Fox et al., 2018; Pecukonis et al., 2021) highlight that atypical brain responses are detectable in ASD infants as early as 6 months, thus indicating that fNIRS might be the an useful tool to early predict ASD.

### Is Hemispheric Asymmetry an Informative Index in fNIRS Recordings?

Theoretical speculations about possible left-hemisphere dysfunction (Fein et al., 1984) or predominant right-hemisphere impairment (Happé, 1999) have historical roots in autism research. Many reports of atypical hemispheric asymmetries in ASD came from anatomical (Herbert et al., 2005) and functional imaging studies (Kleinhans et al., 2008), and typically focused on the abnormal lateralization of language domain (Just et al., 2004), even at very early stages of infants’ development (Eyler et al., 2012). Nevertheless, it is only in the last decade that attention on functional asymmetries related to non-verbal processing and the possibility to consider atypical brain asymmetry as a candidate for clinically meaningful stratification in ASD has grown (Floris et al., 2021). Indeed, recent evidence from resting-state fMRI studies supported the hypothesis that atypical rightward asymmetry shift may be a pervasive feature of functional brain organization in ASD, affecting not only the language network, but also the sensorimotor function and higher cognitive domains (Cardinale et al., 2013). Task-related – fNIRS studies focusing on hemispheric asymmetry in ASD individuals are still very limited and evidence reported are quite inconsistent (Doi and Shinohara, 2017). No systematic findings on hemispheric asymmetry in ASD population during resting-state fNIRS recording are currently available. Even in the studies included in this review, hemispheric asymmetry was not highlighted as a primary research purpose, allowing us to prompt only a few speculations on this topic.

Functional near-infrared spectroscopy studies on language perception and processing seem to confirm previous literature about ASD atypical lateralization for the language domain, with a reduced functional connectivity in the left hemisphere of HR infants compared to the LR peers, both at 6- (Pecukonis et al., 2021) and 12-months of age (Keehn et al., 2013). In contrast, Edwards and colleagues (Edwards et al., 2017) found left- or even hyper-lateralization of neural activity in 3-month-old HR females in response to a language auditory task, speculating about a gender-specific ASD endophenotype related to language processing. However, the limited spatial resolution of fNIRS prevent these results from being conclusive.

Similarly, task-related fNIRS studies of social processing were not consistent in defining hemispheric asymmetry. Indeed, Lloyd-Fox et al. (2013, 2018) found cortical activation patterns in response to social vs. non-social stimuli showing maximal differences between HR and LR groups in the left STS region. Moreover, Braukmann et al. (2018) detected different evoked responses in the right temporal region between groups. In contrast other studies, despite reporting cortical hypoactivation in HR both in naturalistic social interactions (Bhat et al., 2019) and face processing (Fox et al., 2013), did not highlight a clear hemispheric asymmetry of responses.

Finally, resting-state fNIRS studies found a hypoactivation with rightward asymmetry shift in the prefrontal cortical area of ASD preschool children (Li and Yu, 2016; Li et al., 2018). Only two studies reported ASD-related alterations bilaterally (Kikuchi et al., 2013; Jia et al., 2018). However, these results could be influenced by technical aspects, such as the different number of channels used.

Even if no systematic assessment of hemispheric asymmetry has been performed with fNIRS in preschoolers, these findings and evidence from other neuroimaging technique such as structural (Conti et al., 2016) and functional MRI (Müller et al., 2011) would encourage studying more in depth atypical lateralization in ASD population, also at early stage.

### Could fNIRS Be the Right Tool to Dissect the Vascular Contribution to ASD Condition?

Over the last years of research, the biological dimension of ASD has been extensively investigated both in the preclinical (Ebert and Greenberg, 2013; Del Pino et al., 2018) and the clinical settings (Billeci et al., 2016; Bralten et al., 2018). However, most studies only focused on neuronal mechanisms underlying ASD.

Preclinical studies on animal models can offer important insights about specific mechanisms underlying the pathophysiology of ASD (Bartz et al., 2008). Recent studies suggested the presence of structural and functional neurovascular abnormalities in ASD. Indeed, an early dysfunction of endothelial cells, altered angiogenesis and impaired vasodilation reactivity have been shown in a mouse model of 16p11.2 deletion ASD syndrome (Ouellette et al., 2020). Similarly, cerebrovascular deficiencies have been recognized in a mouse model of Creatine Transporter Deficiency, an inherited metabolic condition characterized by intellectual disability and autistic-like features (Mazziotti et al., 2020). The vascular hypothesis is also supported by clinical studies. Indeed, postmortem analysis of ASD brains suggested an impairment of cerebral angiogenesis (Azmitia et al., 2016) and resting-state imaging highlighted the alteration of cerebral blood flow in distinct brain regions (Jann et al., 2015; Bjørklund et al., 2018). Interestingly, it has been speculated that an exacerbated inflammatory response might be one of the possible causes of the deteriorated integrity of the blood-brain barrier (Fiorentino et al., 2016; Kumar and Sharma, 2016) and of the defective blood flow documented in the ASD brain (Morgan et al., 2010; Bjørklund et al., 2018). Moreover, there is evidence of glial dysfunction in ASD (Petrelli et al., 2016), with the alteration of astrocyte population (Fatemi et al., 2008; Edmonson et al., 2014; Wang et al., 2021) potentially contributing to the deregulation of neurovascular homeostasis.

We believe that the potential vascular contribution in the etiology of ASD should be explored more in-depth, since the interplay with the vascular system is crucial for the proper maturation and function of neuronal networks (Andreone et al., 2015; Segarra et al., 2018). In this framework, the combined analysis of hemodynamic responses measured with fNIRS and bioelectrical signals obtained through EEG recordings might allow dissecting vascular vs. neuronal aspects of ASD.

## Conclusion

This review highlights theoretical and methodological advantages of fNIRS that encourage its application for identifying quantitative biomarkers in NDDs, and in particular in the autism field. Even if available papers in preschoolers are yet not conclusive to let us claim a “fNIRS signature” of autism, we believe that the use of this technique as an auxiliary diagnostic tool is very promising. Consistently, we recently reported that the variability of fNIRS visually evoked hemodynamic responses correlates with autistic traits in typically developing children, setting the background for testing the diagnostic value of fNIRS visual measurements in the ASD and HR clinical population (Mazziotti et al., 2022).

Nevertheless, some critical technical issues should be taken into account, because they might limit the interpretation of data and the comparison of results among different studies. First, the availability of reliable methods to detect and remove motion artifacts is fundamental for applying fNIRS in a very young population. Only a few of the studies reviewed (Edwards et al., 2017; Bhat et al., 2019; Pecukonis et al., 2021) systematically applied motion correction algorithms to the data recorded, while most of them simply removed corrupted data (caused by large head or body movements or other unexpected behaviors) by visual inspection of experiment video recordings. This methodological heterogeneity leads not only to a considerable failure rate of fNIRS data collection, but also to a possible bias in the results. Since this topic is debated, wide agreement on subtle technical parameters to apply is still missing even if they could significantly affect data outputs (Di Lorenzo et al., 2019). Moreover, in resting-state measurements complex algorithms to extract reliable data from the fNIRS raw output are often required, representing a potential bias and limiting factor for their clinical use. Indeed, several methodological models have been applied, like functional network efficiency (Li and Yu, 2016, 2018), detrended fluctuation analysis (Jia et al., 2018), coherence analysis of spontaneous hemodynamic fluctuations (Kikuchi et al., 2013) and a combination of principal component analysis and normalized entropy (Li et al., 2018). It is to note that all resting-state studies reviewed compared ASD vs. TD cohorts (3–7-year-old children) and included in the clinical sample only high-functioning autism, probably due to recording constraints (predictable low-grade of compliance during the experimental procedure) that prevent such experimental design in younger and more impaired subjects.

On the other hand, the analysis of articles reviewed in this survey offers several cues of topics to systematically stress in the future fNIRS research within the autism field. Only one of the 13 articles included a focus on the suitability of fNIRS to highlight gender differences in HR infants (Edwards et al., 2017) and the small sample size of other studies (or the lack of a gender-balanced population) did not allow separating gender analyses. However, since consistent findings reported sexual dimorphism of specific brain regions and networks in ASD (see Mo et al., 2021 for a recent review), a specific focus on gender-related brain differences in well-powered cohorts might be an added value of next fNIRS assessments. Similarly, more weight should be given to the extrapolation of asymmetry indexes from fNIRS signals that might represent intra-subject measures potentially useful as condition biomarkers. Moreover, previous studies (Burzi et al., 2015; Jones et al., 2015) explored the importance of imitative learning during early development, focusing on differences in brain activation evoked from live (real time actions) rather than artificial stimuli (videos or photos of actions). While the others used artificial stimuli was applied (Bhat et al., 2019), while in the others artificial stimuli were used. The putative role of stimulus “quality” is still challenging to assess and it might deserve attention in future studies.

Finally, the possibility to measure hemodynamic response with fNIRS would allow the investigation of the possible role of neurovascular coupling in the pathogenesis of ASD, in particular when paired with simultaneous EEG recordings. In this framework, the evolution of hemodynamic patterns from the developing to the adult brain (Kozberg and Hillman, 2016) needs to be considered in the interpretation of typical and aberrant fNIRS signals.

## Author Contributions

EC and ES equally contributed to conceptualization of the manuscript, studies selection, interpretation of results, and manuscript writing. CB contributed to systematic search of the literature. VM, VC, VD, and RM contributed to the conceptualization of the study and interpretation of results. LD, CC, and FM supervised the manuscript. LB contributed to conceptualization, interpretation of technical results, and manuscript writing. SC and RB contributed to conceptualization and supervised the manuscript. All authors have read and agreed the final version of the manuscript.

## Conflict of Interest

The authors declare that the research was conducted in the absence of any commercial or financial relationships that could be construed as a potential conflict of interest.

## Publisher’s Note

All claims expressed in this article are solely those of the authors and do not necessarily represent those of their affiliated organizations, or those of the publisher, the editors and the reviewers. Any product that may be evaluated in this article, or claim that may be made by its manufacturer, is not guaranteed or endorsed by the publisher.
